# The Rising Problem of Hip Fractures in Geriatric Patients—Analysis of Surgical Influences on the Outcome

**DOI:** 10.3390/jpm13081271

**Published:** 2023-08-17

**Authors:** Julian Krickl, Till Ittermann, Andreas Thannheimer, Wolfgang Schmidt, Maximilian Willauschus, Johannes Ruether, Kim Loose, Markus Gesslein, Michael Millrose

**Affiliations:** 1 Department of Trauma Surgery and Sports Medicine, Garmisch-Partenkirchen Medical Centre, 82467 Garmisch-Partenkirchen, Germany; julian.krickl@klinikum-gap.de (J.K.); andreas.thannheimer@klinikum-gap.de (A.T.); wolfgang.schmidt@klinikum-gap.de (W.S.); 2 Institute for Community Medicine, SHIP/Clinical-Epidemiological Research, University of Greifswald, 17489 Greifswald, Germany; till.ittermann@uni-greifswald.de; 3 Department of Orthopedics and Traumatology, Paracelsus Medical University, 90471 Nuremberg, Germany; maximilian.willauschus@klinikum-nuernberg.de (M.W.); johannes.ruether@stud.pmu.ac.at (J.R.); loose.kim@gmail.com (K.L.); markus.gesslein@klinikum-nuernberg.de (M.G.)

**Keywords:** hip fractures, geriatric trauma, orthogeriatrics, frailty, malnutrition

## Abstract

Background: Hip fractures in geriatric patients often have a poor outcome in terms of mortality, mobility as well as independence. Different surgical influence factors are known that improve the outcome. Methods: In this observational cohort study, 281 patients of a geriatric trauma unit were analyzed prospectively. Demographic factors, as well as data regarding the trauma mechanism and perioperative treatment, were recorded. The nutritional status was also analyzed. The follow-up was set to 120 days. Results: The key conclusion of this study is that a high ASA classification, the use of anticoagulatory medicine and malnutrition are significantly associated with higher mortality together with worse independence (*p* < 0.05). There is no significant difference in outcome concerning the time to surgery within the first 24 h. Conclusions: Malnutrition seems to be an important risk factor for an adverse outcome of geriatric patients and therefore warrants a focus in multidisciplinary treatment. The risk factor ASA cannot be improved during the pre-surgery phase, but requires intensified care by a multidisciplinary team specialized in orthogeriatrics.

## 1. Introduction

In a population that is continuously growing older due to the demographic change marked by low birth rates and a rising life expectancy, hip fractures (HF) are among the most critical acute health risk factors in elderly patients. Since more than 90% of hip fractures are accredited to elderly patients over 65 years of age, geriatric trauma surgeons are confronted with preexisting multimorbidity, polymedication and other general geriatric challenges [1]. In Germany in 2021, the incidence of HF in patients 65 years and older is nearly six-fold compared to non-geriatric patients with an additional increased risk of death of up to nineteen-fold [2,3]. The increased mortality risk persists for up to ten years [4]. In a recent prospective study and systematic review, it is clearly depicted that HFs in geriatric patients often cause a considerable deterioration of the functional capacity for both the basic and instrumental activities of daily living. This leads to a significant decline in self-care, daily activities and mobility [5,6]. These fractures also increase the overall mortality of this group of elderly patients [7].

The treatment of HF in elderly patients is most frequently either by total hip arthroplasty or osteosynthesis, and conservative treatment is the exception [8]. Regarding the treatment of HF in geriatric patients, international guidelines, for example, by the American association of orthopedic surgeons, recommend surgery within 24–48 h [9]. These recommendations are based on population-based cohort studies and systematic reviews which show an increased risk of 30-day mortality as well as a poorer outcome [10,11]. Common complications in geriatric patients with HF are infections (systemic or surgery related), perioperative anemia, cognitive impairment (dementia and delirium), metabolic disorders and often subsequent deterioration in mobility and independence [12,13].

Considering this in combination with the estimated global increase in HF from 1.26 million in 1990 to up to 4.5 million in 2050, there is a need to further optimize the treatment and risk evaluation of geriatric patients, since those fractures and their consequences will have an increasing impact on health systems and societies and, consequently, future medical and social costs [14]. Knowing the major risk factors for higher morbidity and mortality might enable physicians to screen patients who are in need of extended diagnostics and special perioperative treatment [15,16].

In previous studies, it could be shown that elderly patients with HF who suffer from malnutrition have increased mortality within the first 12 months and functional dependence over a longer period with the need for more assisted living arrangements, such as nursing homes [17,18].

In this present study, we aim to show the effects of surgical factors, such as time interval to surgery, implant and invasiveness, as well as potential perioperative risk factors, including anemia, dementia or delirium, on mortality and functional outcome. We additionally analyze the impact of preoperative anticoagulation on the outcome and time to surgery and whether malnutrition, assessed by the nutritional risk score (NRS), exerts a higher influence on the outcome than surgical factors.

A secondary analysis of different surgically interesting questions was conducted: whether there is a correlation between the time to surgery and prescribed anticoagulants; whether more invasive surgical techniques require more blood transfusions during the inpatient stay; and whether malnourished geriatric patients show a longer time before surgery due to the need for the preoperative improvement of different blood levels.

## 2. Materials and Methods

This observational cohort study according to the STROBE statement was conducted from January 2018 to December 2020 at a regional care hospital with a department for geriatric trauma surgery [19]. During the entire study period, 1005 elderly patients were admitted to the department with a trauma diagnosis. All geriatric patients with a proximal femoral fracture, regardless of the traumatic or pathological cause, were invited to participate in this study. The traumatic genesis was mostly falls, either because of external reasons, such as slipping or stumbling, or internal reasons, such as syncope or the like. Out of the 1005 admitted patients, all 305 patients with HF were prospectively included in this study. Twenty-four study participants were lost during the follow-up and 281 patients (77 male and 204 female) could be analyzed. Since all patients complying with the inclusion and exclusion criteria could be included, which defined our sample group, the selection bias was minimal.

The inclusion criteria of the study were a minimum age of 65 years and an osteosynthesis of the proximal femur or joint replacement of the hip, regardless of the implant used and whether it was done in an open or closed way. The exclusion criteria of this study were the lack of consent or ability to consent (either themselves or their legal custodians) to the study. Data sets which lack three or more items, regardless of type, were also excluded to minimize the information bias.

The demographics and nutritional status of a part of the study population have already been analyzed and published [18]. The population of this study has been complemented up until December 2020 and focuses on a completely different data set.

This study was conducted in accordance with the guidelines of the declaration of Helsinki. Analysis and publication were permitted by the Institutional Review Board of Paracelsus Medical University (protocol code IRB-2022-018; approved on 23 November 2022). Written informed consent was obtained from every participant or their respective legal custodian.

At inclusion, demographic parameters such as age, sex, preoperative living situation as well as walking ability were recorded. The obtained medical information included the type of fracture classified on a preoperative x-ray, American Society of Anesthesiologists (ASA) classification and prescribed anticoagulatory medication. The time between admission and surgery was logged and categorized into four different categories: <6 h, meaning that the geriatric patient was diagnosed in the emergency department and brought directly to the operation theatre; categories 2 and 3 were 6–12 h and 12–24 h, respectively, because of additional preoperative diagnostics or the lack of OR capacity; and the fourth category of >24 h because of different influence factors. Also, the operation procedure was obtained. Additional to the surgical procedure itself, the invasiveness in terms of closed versus open procedures were differentiated. The outcome parameters were death during the inpatient stay and after 120 days and an alteration in the patient’s living situation and self-dependency, as well as the ability to walk with or without the use of aiding tools. Those changes were acquired at follow-up 120 days after admission.

The preoperative hemoglobin level was routinely obtained. Also, the minimal hemoglobin level of the routine blood works during the inpatient stay of the study participant was analyzed. The need for blood transfusion and the amount of packed red blood cells (pRBCs) as well as the need for antibiotic therapy and whether it was necessary because of surgery-related complications or other infections were recorded. The presence of dementia was assessed either anamnestically or with a mini-mental state examination [20]. Also, the occurrence of postoperative delirium was included in the analysis.

To assess the nutritional status of the included geriatric patients, the body mass index (BMI) and nutritional risk score were analyzed. The nutritional risk score, a simple and well-validated tool which is one of the most often used screening tools in hospitals worldwide, was recorded during the inpatient stay by nutritionists [21]. A total score of ≥3 points means that the patient is at risk of malnutrition or already malnourished and, therefore, a nutritional therapy might be necessary.

The data were acquired prospectively and analyzed retrospectively. The statistical analysis was completed by using STATA 17.0 (Stata Corporation, College Station, TX, USA). Features of the study population are reported and stratified by sex as the mean and standard deviation for continuous variables and as absolute numbers and percentages for categorical variables. Associations of potential risk factors with inpatient death, mortality after 120 days, worsened mobility after surgery and worsened living situation postoperatively were analyzed by logistic regression models according to the Wald Chi-squared test. Those models were adjusted for age and sex. The results are reported as odds ratio, 95% confidence interval and *p*-value. A *p* < 0.05 was considered as statistically significant. The time between admission and surgery was categorized into quarters of the first 24 h. The invasiveness and implant were categorized into procedures with bigger skin incisions and exposure as “open” versus percutaneous procedures as “closed” in order to detect the influence of additional iatrogenic trauma.

## 3. Results

During this prospective observational cohort study, 281 geriatric patients were included and analyzed. They sustained hip or proximal femur fractures and were admitted and treated at the geriatric trauma surgery department of a regional trauma center. Of the 281 study patients included, 204 were female with a mean age of 84.5 ± 7.2 years. The 77 men had a mean age of 83.53 ± 5.92 years. The measurements of the geriatric patients can be found in the supplementary material (Table S1). The BMIs of the included female and male patients were 23.69 ± 4.06 and 24.33 ± 3.4, respectively. Concerning the perioperative risk depicted in the ASA classification, 3 were categorized as healthy patients with ASA 1, 50 had mild systemic disease (ASA 2), 203 had severe systemic diseases (ASA 3) and 25 were constantly threatened by death because of their systemic disease-ASA 4.

During the phase of inpatient treatment, 25 patients died, while at the follow-up that occurred 120 days after surgery, the number increased to 51 patients. The details of the accessory outcome parameters (mobility and independence at follow-up) are shown in Table 1.

The level of mobility of the 230 patients available at follow-up was improved or stayed the same in comparison to their preadmission level in 114 patients, while 116 patients suffered from a worse mobility level, namely those who needed additional walking support or were only mobile indoors and needed a wheelchair for outside.

With respect to the independence of the living situation, 6 patients could live in a more self-reliant manner, 201 lived as before and 23 lost their ability to live independently and were cared for in nursing homes or in a hospital.

The data of the analyzed surgical risk factors and malnutrition are depicted in Table 2 in detail. The included patients of this study groups solely used phenprocoumon as an oral anticoagulant (OAC) and Apixaban, Edoxaban or Rivaroxaban as a novel oral anticoagulant (NOAC). The prescribed platelet aggregation inhibitors were either acetylsalicylic acid or Clopidogrel.

The results of the regression analysis are shown in Table 3. The analysis shows that the preoperative prescription of an anticoagulant increases mortality during the inpatient stay. OACs especially show a highly elevated risk of death during the clinical stay as well as during the follow-up, whereas PAIs do not seem to have an influence on mortality. As expected, anticoagulation has no effect on mobility and independence. The data show that severely ill geriatric patients with ASA 4, and to a lesser degree, also ASA 3, significantly worsens the outcome with respect to mortality as well as independence. The surgical technique and implant used had a significant influence on mobility. The open surgical technique used in 64% of patients with THR improved mobility at the follow-up occurring 120 days after surgery. A preoperative anemia is associated with higher mortality and worsened mobility at follow-up. The minimal hemoglobin level during the whole inpatient stay had no significant effect on the outcome parameters. In concordance, the amount of transfused pRBCs shows a similar correlation with mortality at follow-up, similar to the preoperative hemoglobin level. The nutritional status of the geriatric patient, as reflected by a lower BMI and higher NRS, shows significantly higher mortality during inpatient stay and at follow-up, as well as worsened independence.

The secondary analysis showed that there was no significant influence of a prescribed anticoagulation medication and the time between admission and surgery (*p* = 0.76 OAC; *p* = 0.350 PAI; *p* = 0.559 NOAC). Also, there was no significant correlation between the invasiveness of the surgical technique and the need for pRBCs (*p* = 0.704). The malnutrition of the geriatric patient had no significant effect on the time to surgery after admission (*p* = 0.153).

## 4. Discussion

The results of this study show different statistically significant risk factors for a worse outcome after HF in geriatric patients. Interestingly, the highest influence could be established for pre-trauma conditions, namely the use of anticoagulant medication, morbidity because of underlying health conditions (depicted in the ASA classification), as well as malnutrition. All the above-mentioned influence factors are typically present in a growing elderly patient population; therefore, they should move into the focus of geriatric trauma surgeons.

In our study, we could show that pre-surgery anemia is correlated with higher mortality and a worsened outcome in terms of mobility and independence. This is in concordance with recently published studies [22,23]. Also, elderly patients are much more likely to receive an anticoagulatory medication, which as an influence factor itself, increases mortality during inpatient stay and might also increase the severity of blood loss from the fracture, both during surgery and postoperatively. In a systematic review, it could be shown that pre-surgery anemia is an independent risk factor for surgery-related infections [24]. Hansen et al. suggested to treat the preexisting anemia with intravenous iron therapy during the inpatient stay, but could not show an effect on surgery-related infections [25]. A Cochrane systematic review from 2015 regarding the transfusion of pRBCs for patients undergoing HF surgery could show no beneficiary effect of a liberal transfusion strategy in terms of mortality [26]. Based on the results of our study, this conclusion might need reconsideration since the mortality, mobility and independence of geriatric patients are all affected. Nevertheless, our results showed, significantly, that the need for a transfusion and the amount of pRBCs during the inpatient stay increases mortality at follow-up, while the minimal hemoglobin level only showed trends, which is in concordance with a recently published study by Garg et al. [27]. Since we apply a restrictive transfusion strategy in our geriatric trauma unit, the patients most likely showed clinical signs of anemia before they were transfused, which is probably because of the comorbidities reflected in their ASA classification.

As one would expect, the data of this prospective study showed that a higher ASA risk classification of the geriatric patient dramatically increases the rate of death during the inpatient stay and after 120 days at follow-up. This is comparable to the results of Haugn et al. and Quach et al., who could show that multimorbidity, depicted by a higher ASA risk score, might lead to higher mortality 30 days and 1 year after HF, respectively [28,29]. Cher et al. even postulated that comorbidities are the dominant influence factor of mortality after HF in the geriatric patient [30]. These findings might lead to the impression that severely ill geriatric patients with HF should be treated conservatively to spare them the perioperative risks. Different systematic reviews and meta-analysis clearly show that the nonoperative treatment of those patients results in a poor prognosis with higher complication rates and mortality [8,31]. Therefore, the surgical treatment of HF in geriatric patients is strongly recommended in international guiding principles [9].

There is clear evidence in the literature that a short time to surgery has a beneficial effect on short- and long-term mortality [32,33]. An early surgery is also associated with a reduced pRBC transfusion rate [34]. Those studies define an early surgery by time to surgery of less than 48 h. The results of our study are in contrast since we were not able to show an influence of time to surgery with statistical significance. There might be different reasons. In a recent study by Fenwick et al., the authors reported that short-term mortality did not increase during the first 48 h of inpatient stay [35]. Since we categorized the time to surgery only during the first 24 h, the differences in between might be too subtle to be detected within the included patient collective. Another aspect is that a perioperative optimization of the patient, for example, in terms of electrolyte imbalances, can improve the outcome [36,37]. Since most of the preoperative interventions are completed in the emergency department, they most likely take place during the first 6-12 h with a developing effect on the geriatric patient within 24 h. Therefore, the mortality of those geriatric patients in need of optimization might decrease during the first 24 h. There is the need for an evaluation of the specific preoperative optimization within our study collective in order to substantiate this attempt of explanation.

Our study showed that prescribed oral anticoagulatory medicine, especially vitamin k antagonists, increases the risk for mortality during the inpatient stay and follow-up. This is in concordance with a prospective cohort study by Blanco et al. in 2021, who analyzed the influence of preoperative clinical variables on 30-day mortality [38]. One reason could be that particularly patients who suffer from chronic cardiac insufficiency or have a previous diagnosis of arrhythmia or previous history of ischemic heart failure usually take anticoagulant treatment. This special group of elderly patients with cardiovascular disease are at higher risk of dying, which Cha et al. demonstrated in 2019 [39]. In the studies by Blanco et al. as well as Rutenberg et al. in 2018, the use of the vitamin K antagonist was associated with a longer time to surgery, which both authors offered as a reason for increased mortality [38,40]. Interestingly, we could not show any statistically significant association between anticoagulatory medicine and time between admission and surgery. This might be due to the short time intervals defined in our study.

In accordance with our study on the effect of the malnutrition of elderly patients on mortality and mobility, we could show the same effect in this patient collective [18]. Recent systematic reviews find a high prevalence of malnutrition amongst elderly patients and support the beneficial role of oral nutrition supplements [41,42]. Gonul et al. showed in their recent study that malnutrition increases the odds of mortality 30 days after surgery by 4.2 times, which is in concordance with our findings of an increased risk of dying during the inpatient stay of 6.7 and at 120 days after surgery by 3.3 [43]. A Cochrane review produced evidence that a multidisciplinary rehabilitation, including the optimization of nutrition, resulted in fewer cases of poor outcome in terms of mortality and independence [44]. Since malnutrition is a risk factor which is accessible for treatment during inpatient stay and rehabilitation treatment, this should be paid attention to by responsible physicians.

One of the strengths of our study is the prospective study design of 281 consecutive patients treated at a geriatric trauma department for HF. During the inclusion period, no essential changes in therapy were made. Also, the study population shows a high percentage of patients (85%) who were surgically treated within the first 24 h, which enables this study to further analyze the early time span of therapy, while most of the published studies differentiate between less or more than 48 h [32,33]. Additionally, concerning the important risk factor of malnutrition, the NRS in our study was obtained by nutritionists. Benoit et al. were able to show in a clinical study that the assessment of the nutritional status by surgeons was significantly worse than by nutritionists [45]. Therefore, the nutritional data acquired in our study represent the real status of the included patient more accurately.

One limitation of this study is the follow-up of only 120 days. Peeters et al. showed in a systematic review that the outcome of elderly patients after HF can improve during the first 6 months; therefore, the reported outcomes in terms of mortality or mobility and to a lesser degree, independence, might be worse after 6 months or longer of rehabilitation [5]. Confounding diagnoses likely to be present in geriatric patients, such as chronic obstructive pulmonary disease, cardiovascular disease or diabetes, were not analyzed and could have an effect on the outcome after the HF of the elderly patient. The blood iron level and the type of pre-surgery anemia should have been obtained in order to differentiate perioperative blood loss from iron-deficiency anemia.

## 5. Conclusions

Geriatric patients with HF are at high risk of experiencing an adverse outcome. Besides risk factors that cannot be altered pre-surgically by interdisciplinary treatment, such as the health status of the patients depicted in the ASA classification, malnutrition seems to be an important influence factor on the outcome, which should be treated during the inpatient stay and rehabilitation. Since prescribed anticoagulatory medication also shows a correlation to higher mortality, the perioperative therapy in terms of antidotes or supplementation should gain the appropriate attention.

## Figures and Tables

**Table 1:** Details of the additional outcome parameters: mobility and independence at follow-up.

Mobility	Independent	Cane/Crutch	Rollator	Only Indoor	Bedridden
Preadmission [n = 281]	97	31	66	76	11
Follow-up 120d [n = 230]	36	30	79	58	27
**Independence**	**Own home**	**Nursing home**	**Hospital**		
Preadmission [n = 281]	221	60			
Follow-up 120d [n = 230]	177	51	2		

**Table 2:** Details of the different analyzed surgical risk factors.

	Female (n = 204)	Male (n = 77)	Total (n = 281)
**Anticoagulant**			
None	104 (51.0%)	28 (36.4%)	132 (47.0%)
OAC	13 (6.4%)	8 (10.4%)	21 (7.5%)
PAI	46 (22.5%)	17 (22.1%)	63 (22.4%)
NOAC	37 (18.1%)	15 (19.5%)	52 (18.5%)
combination	4 (2.0%)	9 (11.7%)	13 (4.6%)
**Any anticoagulant use**	96 (47.1%)	40 (51.9%)	136 (48.4%)
**Time between admission and surgery**	16.1 (29.0)	15.0 (21.9)	15.8 (27.2)
<6 h	78 (38.2%)	25 (32.5%)	103 (36.7%)
6 to 12 h	31 (15.2%)	18 (23.4%)	49 (17.4%)
12 to 24 h	63 (30.9%)	23 (29.9%)	86 (30.6%)
>24 h	32 (15.7%)	11 (14.3%)	43 (15.3%)
**Invasiveness**			
Closed	96 (47.1%)	42 (54.5%)	138 (49.1%)
Open	108 (52.9%)	35 (45.5%)	143 (50.9%)
**Implant**			
DHS or nail (CRIF)	103 (50.5%)	46 (59.7%)	149 (53.0%)
Nail (ORIF) or THR	88 (43.1%)	30 (39.0%)	118 (42.0%)
Osteosynthesis	13 (6.4%)	1 (1.3%)	14 (5.0%)
**Hemoglobin preoperative [g/dL]**	12.5 (1.7)	12.4 (2.0)	12.5 (1.8)
**Hemoglobin minimal [g/dL]**	8.2 (1.4)	8.4 (1.6)	8.3 (1.5)
**Transfusion**	80 (39.2%)	27 (35.1%)	107 (38.1%)
**Amount of transfused pRBC**	1.9 (1.0)	1.9 (1.2)	1.9 (1.0)
**Antibiotic**	82 (40.2%)	29 (37.7%)	111 (39.5%)
**Dementia**	126 (62.1%)	38 (50.0%)	164 (58.8%)
**Delirium**	24 (11.8%)	12 (15.8%)	36 (12.9%)
**NRS**			
<3	53 (40.2%)	26 (49.1%)	79 (42.7%)
≥3	79 (59.8%)	27 (50.9%)	106 (57.3%)

OAC: oral anticoagulants; PAI: platelet aggregation inhibitors; NOAC: novel oral anticoagulants; DHS: dynamic hip screw; nail (CRIF): closed reduction and internal fixation with trochanteric nail; nail (ORIF): open reduction and internal fixation with trochanteric nail; THR: total hip replacement; NRS: nutritional risk score; categorial data are shown as absolute numbers and percentages.

**Table 3:** Associations of surgical risk factors with mortality during inpatient stay or at follow-up and worsened mobility or domicile (n = 281).

	Mortality Inpatient OR (95%-CI)	Mortality at 120 d OR (95%-CI)	Worsened Mobility OR (95%-CI)	Worsened Domicile OR (95%-CI)
**Any anticoagulant use** OAC vs. none PAI vs. none NOAC vs. none	3.16 (1.23; 8.15) * 9.28 (2.29; 37.6) * 1.76 (0.44; 7.04) 4.95 (1.46; 16.7) *	1.84 (0.97; 3.49) 3.00 (1.01; 8.90) * 1.59 (0.69; 3.65) 1.99 (0.86; 4.63)	1.17 (0.72; 1.90) 1.40 (0.52; 3.75) 1.30 (0.69; 2.42) 0.99 (0.52; 1.92)	1.24 (0.72; 2.14) 1.82 (0.67; 4.98) 1.00 (0.49; 2.07) 1.41 (0.68; 2.94)
**ASA risk classification** 3 vs. (1,2) 4 vs. (1,2)	3.16 (0.40; 25.2) 15.7 (1.73; 142) *	10.0 (1.33; 75.6) * 29.2 (3.39; 251) *	0.95 (0.50; 1.78) 1.26 (0.45; 3.54)	2.45 (0.97; 6.17) 5.14 (1.57; 16.9) *
**Time between admission and surgery** 6 to 12 h vs. <6 h 12 to 24 h vs. <6 h	1.74 (0.57; 5.27) 0.95 (0.30; 2.95)	1.27 (0.53; 3.00) 1.04 (0.48; 2.26)	1.10 (0.53; 2.29) 0.61 (0.34; 1.10)	1.02 (0.47; 2.22) 1.02 (0.53; 1.98)
>24 h vs. <6 h	0.75 (0.18; 3.21)	0.72 (0.26; 2.05)	0.82 (0.39; 1.72)	0.59 (0.24; 1.48)
**Invasiveness** Open vs. closed	2.07 (0.84; 5.11)	1.77 (0.93; 3.39)	0.57 (0.35; 0.93) *	1.22 (0.70; 2.12)
**Implant** (THR, nail ORIF) vs. (DHS, nail CRIF)	1.26 (0.52; 3.04)	1.40 (0.73; 2.70)	0.52 (0.32: 0.87) *	1.02 (0.58; 1.80)
**Body mass index [kg/m^2^]**	0.93 (0.81; 1.06)	0.90 (0.82; 0.99) *	1.00 (0.94; 1.06)	0.94 (0.87; 1.02)
**Hemoglobin preoperative [g/dL]**	0.88 (0.70; 1.11)	0.79 (0.66; 0.94) *	0.84 (0.72; 0.97) *	0.84 (0.72; 0.98) *
**Hemoglobin minimal [g/dL]**	0.72 (0.52; 1.01)	0.86 (0.69; 1.08)	0.88 (0.75; 1.04)	0.89 (0.73; 1.08)
**Transfusion**	1.32 (0.55; 3.15)	1.91 (1.01; 3.62) *	1.47 (0.88; 2.43)	1.61 (0.92; 2.81)
**Amount of transfused pRBCs**	1.28 (0.68; 2.42)	1.77 (1.11; 2.83) *	1.37 (0.88; 2.15)	1.57 (1.01; 2.43) *
**Antibiotics use**	0.83 (0.34; 2.03)	1.34 (0.71; 2.52)	1.21 (0.74; 1.98)	1.43 (0.82; 2.50)
**Dementia**	0.65 (0.24; 1.76)	0.92 (0.43; 1.98)	0.95 (0.54; 1.68)	1.15 (0.59; 2.25)
**Delirium**	0.16 (0.02; 1.38)	0.92 (0.32; 2.64)	1.11 (0.48; 2.56)	1.08 (0.42; 2.74)
**Nutritional risk score** ≥3 vs. <3	6.69 (1.39; 32.1) *	3.30 (1.30; 8.36)*	1.44 (0.78; 2.66)	2.68 (1.25; 5.76) *

Logistic regression models adjusted for age and sex, OR: odds ratio; CI: confidence interval; statistical significance level * *p* < 0.05.; green: significantly lower risk for adverse event; red: significantly higher risk for adverse event. OAC: oral anticoagulants; PAI: platelet aggregation inhibitors; NOAC: novel oral anticoagulants; DHS: dynamic hip screw; nail (CRIF): closed reduction and internal fixation with trochanteric nail; nail (ORIF): open reduction and internal fixation with trochanteric nail; THR: total hip replacement.

## Data Availability

The data of the study population are provided in the Supplementary Material Table S1. The complete data of the regression analysis depicted in Table 3 are available in the supplementary material complete regression analysis results (Table 3).

## Supplementary Materials

The following supporting information can be downloaded at: https://www.mdpi.com/article/10.3390/jpm13081271/s1, Table S1: Data of included patients.
