# Supplementary material for: The Rising Problem of Hip Fractures in Geriatric Patients—Analysis of Surgical Influences on the Outcome
Source: J Pers Med. 2023 Aug 17;13(8):1271. doi: 10.3390/jpm13081271 (PMC10455730; doi:10.3390/jpm13081271)
Supplement: Supplementary file 1 [file jpm-13-01271-s001.zip › Supplementary Data Complete regression analysis results (Table 3).docx]

Exposure: Any Anticoagulant; Outcome: Mortality inpatient

Logistic regression Number of obs = 281

LR chi2(3) = 27.71

Prob > chi2 = 0.0000

Log likelihood = -70.487297 Pseudo R2 = 0.1643

------------------------------------------------------------------------------

death_inc | Odds ratio Std. err. z P>|z| [95% conf. interval]

-------------+----------------------------------------------------------------

1.anyanticoa | 3.162294 1.528433 2.38 0.017 1.226283 8.15481

Age | 1.145428 .0444929 3.50 0.000 1.06146 1.236038

|

sex |

male | 4.577659 2.168471 3.21 0.001 1.808935 11.58414

_cons | 2.19e-07 7.79e-07 -4.31 0.000 2.06e-10 .0002335

------------------------------------------------------------------------------

Note: _cons estimates baseline odds.

Exposure: Any Anticoagulant; Outcome: Mortality at 120 d

Logistic regression Number of obs = 281

LR chi2(3) = 21.55

Prob > chi2 = 0.0001

Log likelihood = -122.3196 Pseudo R2 = 0.0810

------------------------------------------------------------------------------

death120 | Odds ratio Std. err. z P>|z| [95% conf. interval]

-------------+----------------------------------------------------------------

1.anyanticoa | 1.837887 .6004775 1.86 0.062 .9687522 3.486782

Age | 1.089632 .0275418 3.40 0.001 1.036966 1.144972

|

sex |

male | 2.516433 .8556887 2.71 0.007 1.292238 4.900363

_cons | .0000775 .0001735 -4.23 0.000 9.66e-07 .0062222

------------------------------------------------------------------------------

Note: _cons estimates baseline odds.

Exposure: Any Anticoagulant; Outcome: Worsened mobility

Logistic regression Number of obs = 281

LR chi2(3) = 7.46

Prob > chi2 = 0.0586

Log likelihood = -186.01671 Pseudo R2 = 0.0197

---------------------------------------------------------------------------------

mobility_change | Odds ratio Std. err. z P>|z| [95% conf. interval]

----------------+----------------------------------------------------------------

1.anyanticoa | 1.173738 .2896014 0.65 0.516 .7236868 1.903671

Age | 1.036489 .0186684 1.99 0.047 1.000538 1.073732

|

sex |

male | 1.660462 .4717696 1.78 0.074 .9514499 2.897823

_cons | .0582388 .0889035 -1.86 0.063 .002923 1.160381

---------------------------------------------------------------------------------

Note: _cons estimates baseline odds.

Exposure: Any Anticoagulant; Outcome: Worsened domicile

Logistic regression Number of obs = 281

LR chi2(3) = 16.96

Prob > chi2 = 0.0007

Log likelihood = -153.52536 Pseudo R2 = 0.0523

---------------------------------------------------------------------------------

domicile_change | Odds ratio Std. err. z P>|z| [95% conf. interval]

----------------+----------------------------------------------------------------

1.anyanticoa | 1.236994 .345811 0.76 0.447 .7151674 2.139574

Age | 1.081201 .023108 3.65 0.000 1.036845 1.127454

|

sex |

male | 1.698727 .5159755 1.74 0.081 .9366504 3.080843

_cons | .0003586 .0006682 -4.26 0.000 9.31e-06 .013821

---------------------------------------------------------------------------------

Note: _cons estimates baseline odds.

Exposure: Anticoagulant; Outcome: Mortality inpatient

Logistic regression Number of obs = 281

LR chi2(6) = 34.59

Prob > chi2 = 0.0000

Log likelihood = -67.044569 Pseudo R2 = 0.2051

-------------------------------------------------------------------------------

death_inc | Odds ratio Std. err. z P>|z| [95% conf. interval]

--------------+----------------------------------------------------------------

anticoagulant |

OAC | 9.37913 6.759106 3.11 0.002 2.284236 38.51095

ASS/Plavix | 1.775106 1.260551 0.81 0.419 .4413292 7.139796

NOAC | 5.054287 3.158166 2.59 0.010 1.485209 17.20015

combination | 3.737755 3.609474 1.37 0.172 .5631485 24.8084

|

Age | 1.150742 .046898 3.45 0.001 1.062399 1.246431

|

sex |

male | 3.697148 1.819241 2.66 0.008 1.409352 9.698713

_cons | 1.27e-07 4.75e-07 -4.25 0.000 8.35e-11 .0001931

-------------------------------------------------------------------------------

Note: _cons estimates baseline odds.

Exposure: Anticoagulant; Outcome: Mortality at 120 d

Logistic regression Number of obs = 281

LR chi2(6) = 23.00

Prob > chi2 = 0.0008

Log likelihood = -121.59838 Pseudo R2 = 0.0864

-------------------------------------------------------------------------------

death120 | Odds ratio Std. err. z P>|z| [95% conf. interval]

--------------+----------------------------------------------------------------

anticoagulant |

OAC | 2.976376 1.668606 1.95 0.052 .9919502 8.930704

ASS/Plavix | 1.601077 .6816227 1.11 0.269 .6950763 3.688011

NOAC | 1.999749 .8658695 1.60 0.109 .8558835 4.672362

combination | 1.611928 1.20542 0.64 0.523 .3722259 6.98047

|

Age | 1.089681 .0277878 3.37 0.001 1.036557 1.145528

|

sex |

male | 2.331967 .8160991 2.42 0.016 1.17445 4.630313

_cons | .000075 .0001695 -4.20 0.000 8.93e-07 .0062968

-------------------------------------------------------------------------------

Note: _cons estimates baseline odds.

Exposure: Anticoagulant; Outcome: Worsened mobility

Logistic regression Number of obs = 281

LR chi2(6) = 8.08

Prob > chi2 = 0.2321

Log likelihood = -185.7046 Pseudo R2 = 0.0213

---------------------------------------------------------------------------------

mobility_change | Odds ratio Std. err. z P>|z| [95% conf. interval]

----------------+----------------------------------------------------------------

anticoagulant |

OAC | 1.3785 .6950955 0.64 0.524 .5130937 3.70354

ASS/Plavix | 1.289641 .4114788 0.80 0.425 .6900496 2.410222

NOAC | .985176 .3308319 -0.04 0.965 .5101194 1.902636

combination | .9878556 .6107301 -0.02 0.984 .2940682 3.318477

|

Age | 1.036634 .0187082 1.99 0.046 1.000607 1.073957

|

sex |

male | 1.660266 .4848757 1.74 0.083 .9366706 2.942853

_cons | .0576244 .0881994 -1.86 0.062 .0028692 1.157305

---------------------------------------------------------------------------------

Note: _cons estimates baseline odds.

Exposure: Anticoagulant; Outcome: Worsened domicile

Logistic regression Number of obs = 281

LR chi2(6) = 18.43

Prob > chi2 = 0.0052

Log likelihood = -152.78737 Pseudo R2 = 0.0569

---------------------------------------------------------------------------------

domicile_change | Odds ratio Std. err. z P>|z| [95% conf. interval]

----------------+----------------------------------------------------------------

anticoagulant |

OAC | 1.826748 .9408801 1.17 0.242 .6656727 5.012984

ASS/Plavix | 1.005504 .3723891 0.01 0.988 .4865652 2.077911

NOAC | 1.419893 .5321045 0.94 0.350 .6811901 2.959669

combination | 1.315422 .8764399 0.41 0.681 .3563908 4.855165

|

Age | 1.08129 .0232714 3.63 0.000 1.036627 1.127877

|

sex |

male | 1.612135 .5051629 1.52 0.127 .8723227 2.979378

_cons | .0003518 .0006608 -4.23 0.000 8.86e-06 .0139699

---------------------------------------------------------------------------------

Note: _cons estimates baseline odds.

Exposure: ASA risk classification; Outcome: Mortality inpatient

Logistic regression Number of obs = 281

LR chi2(4) = 32.25

Prob > chi2 = 0.0000

Log likelihood = -68.213763 Pseudo R2 = 0.1912

------------------------------------------------------------------------------

death_inc | Odds ratio Std. err. z P>|z| [95% conf. interval]

-------------+----------------------------------------------------------------

asa |

3 | 3.156597 3.344322 1.08 0.278 .3957271 25.17923

4 | 15.68673 17.66054 2.45 0.014 1.726741 142.5075

|

Age | 1.125944 .0447185 2.99 0.003 1.041622 1.217093

|

sex |

male | 4.501112 2.188363 3.09 0.002 1.735723 11.67238

_cons | 5.24e-07 1.93e-06 -3.92 0.000 3.77e-10 .0007276

------------------------------------------------------------------------------

Note: _cons estimates baseline odds.

Exposure: ASA risk classification; Outcome: Mortality at 120 d

Logistic regression Number of obs = 281

LR chi2(4) = 35.32

Prob > chi2 = 0.0000

Log likelihood = -115.4385 Pseudo R2 = 0.1327

------------------------------------------------------------------------------

death120 | Odds ratio Std. err. z P>|z| [95% conf. interval]

-------------+----------------------------------------------------------------

asa |

3 | 10.02122 10.33108 2.24 0.025 1.328616 75.58602

4 | 29.17817 32.03832 3.07 0.002 3.391709 251.0137

|

Age | 1.074195 .0284788 2.70 0.007 1.019803 1.131488

|

sex |

male | 2.362739 .8255242 2.46 0.014 1.191275 4.686185

_cons | .0000392 .0000982 -4.05 0.000 2.90e-07 .0052975

------------------------------------------------------------------------------

Note: _cons estimates baseline odds.

Exposure: ASA risk classification; Outcome: Worsened mobility

Logistic regression Number of obs = 281

LR chi2(4) = 7.44

Prob > chi2 = 0.1144

Log likelihood = -186.0267 Pseudo R2 = 0.0196

---------------------------------------------------------------------------------

mobility_change | Odds ratio Std. err. z P>|z| [95% conf. interval]

----------------+----------------------------------------------------------------

asa |

3 | .947443 .3052924 -0.17 0.867 .5038165 1.781697

4 | 1.259068 .6636478 0.44 0.662 .4481106 3.537634

|

Age | 1.035879 .0191428 1.91 0.056 .9990315 1.074086

|

sex |

male | 1.670351 .4770057 1.80 0.072 .9543951 2.923393

_cons | .0672861 .1032091 -1.76 0.079 .0033287 1.360096

---------------------------------------------------------------------------------

Note: _cons estimates baseline odds.

Exposure: ASA risk classification; Outcome: Worsened domicile

Logistic regression Number of obs = 281

LR chi2(4) = 24.34

Prob > chi2 = 0.0001

Log likelihood = -149.83158 Pseudo R2 = 0.0751

---------------------------------------------------------------------------------

domicile_change | Odds ratio Std. err. z P>|z| [95% conf. interval]

----------------+----------------------------------------------------------------

asa |

3 | 2.453325 1.155168 1.91 0.057 .9748995 6.173768

4 | 5.138579 3.116918 2.70 0.007 1.565049 16.87167

|

Age | 1.071044 .0235858 3.12 0.002 1.025801 1.118284

|

sex |

male | 1.602251 .4943061 1.53 0.127 .8752433 2.933138

_cons | .0003874 .0007446 -4.09 0.000 8.96e-06 .0167532

---------------------------------------------------------------------------------

Note: _cons estimates baseline odds.

Exposure: Time between Admission and Surgery in hours; Outcome: Mortality inpatient

Logistic regression Number of obs = 281

LR chi2(3) = 22.87

Prob > chi2 = 0.0000

Log likelihood = -72.907026 Pseudo R2 = 0.1356

--------------------------------------------------------------------------------

death_inc | Odds ratio Std. err. z P>|z| [95% conf. interval]

---------------+----------------------------------------------------------------

admisssurgtime | .9860635 .0155423 -0.89 0.373 .9560668 1.017001

Age | 1.144427 .0439308 3.51 0.000 1.061484 1.233852

|

sex |

male | 4.664555 2.182427 3.29 0.001 1.864466 11.66987

_cons | 5.65e-07 1.96e-06 -4.15 0.000 6.32e-10 .0005055

--------------------------------------------------------------------------------

Note: _cons estimates baseline odds.

Exposure: Time between Admission and Surgery in hours; Outcome: Mortality at 120 d

Logistic regression Number of obs = 281

LR chi2(3) = 18.01

Prob > chi2 = 0.0004

Log likelihood = -124.08947 Pseudo R2 = 0.0677

--------------------------------------------------------------------------------

death120 | Odds ratio Std. err. z P>|z| [95% conf. interval]

---------------+----------------------------------------------------------------

admisssurgtime | 1.000339 .0054663 0.06 0.951 .9896828 1.011111

Age | 1.088198 .0272115 3.38 0.001 1.036151 1.142861

|

sex |

male | 2.540973 .8566462 2.77 0.006 1.312296 4.920036

_cons | .0001192 .0002611 -4.12 0.000 1.63e-06 .0087264

--------------------------------------------------------------------------------

Note: _cons estimates baseline odds.

Exposure: Time between Admission and Surgery in hours; Outcome: Worsened mobility

Logistic regression Number of obs = 281

LR chi2(3) = 7.24

Prob > chi2 = 0.0645

Log likelihood = -186.12426 Pseudo R2 = 0.0191

---------------------------------------------------------------------------------

mobility_change | Odds ratio Std. err. z P>|z| [95% conf. interval]

----------------+----------------------------------------------------------------

admisssurgtime | .9979517 .0044823 -0.46 0.648 .989205 1.006776

Age | 1.037173 .0186889 2.03 0.043 1.001183 1.074457

|

sex |

male | 1.669717 .4738579 1.81 0.071 .9573611 2.912124

_cons | .0613795 .093484 -1.83 0.067 .0031017 1.21463

---------------------------------------------------------------------------------

Note: _cons estimates baseline odds.

Exposure: Time between Admission and Surgery in hours; Outcome: Worsened domicile

Logistic regression Number of obs = 281

LR chi2(3) = 16.74

Prob > chi2 = 0.0008

Log likelihood = -153.63524 Pseudo R2 = 0.0517

---------------------------------------------------------------------------------

domicile_change | Odds ratio Std. err. z P>|z| [95% conf. interval]

----------------+----------------------------------------------------------------

admisssurgtime | .9968092 .0055679 -0.57 0.567 .9859558 1.007782

Age | 1.082151 .0231873 3.68 0.000 1.037646 1.128565

|

sex |

male | 1.714035 .5201333 1.78 0.076 .9456226 3.106858

_cons | .0003877 .0007199 -4.23 0.000 .0000102 .0147604

---------------------------------------------------------------------------------

Note: _cons estimates baseline odds.

Exposure: Time between Admission and Surgery in hours; Outcome: Mortality inpatient

Logistic regression Number of obs = 281

LR chi2(5) = 23.14

Prob > chi2 = 0.0003

Log likelihood = -72.772462 Pseudo R2 = 0.1372

------------------------------------------------------------------------------

death_inc | Odds ratio Std. err. z P>|z| [95% conf. interval]

-------------+----------------------------------------------------------------

timeadsu_cat |

<12h | 1.737583 .9834553 0.98 0.329 .5730248 5.26887

<24h | .9471605 .5490378 -0.09 0.925 .3040955 2.950102

> 24h | .7525608 .5566638 -0.38 0.701 .1765688 3.207519

|

Age | 1.134251 .0430849 3.32 0.001 1.052873 1.221919

|

sex |

male | 4.333814 2.036206 3.12 0.002 1.725601 10.88429

_cons | 9.71e-07 3.33e-06 -4.03 0.000 1.16e-09 .0008102

------------------------------------------------------------------------------

Note: _cons estimates baseline odds.

Exposure: Time between Admission and Surgery in hours; Outcome: Mortality at 120 d

Logistic regression Number of obs = 281

LR chi2(5) = 18.99

Prob > chi2 = 0.0019

Log likelihood = -123.60259 Pseudo R2 = 0.0713

------------------------------------------------------------------------------

death120 | Odds ratio Std. err. z P>|z| [95% conf. interval]

-------------+----------------------------------------------------------------

timeadsu_cat |

<12h | 1.265222 .5572511 0.53 0.593 .5336611 2.99963

<24h | 1.037559 .4109766 0.09 0.926 .477367 2.255139

> 24h | .7249886 .3849759 -0.61 0.545 .256056 2.052709

|

Age | 1.087712 .0274074 3.34 0.001 1.0353 1.142778

|

sex |

male | 2.513013 .8551478 2.71 0.007 1.289855 4.896077

_cons | .0001232 .0002736 -4.05 0.000 1.59e-06 .0095639

------------------------------------------------------------------------------

Note: _cons estimates baseline odds.

Exposure: Time between Admission and Surgery in hours; Outcome: Worsened mobility

Logistic regression Number of obs = 281

LR chi2(5) = 10.64

Prob > chi2 = 0.0589

Log likelihood = -184.4238 Pseudo R2 = 0.0280

---------------------------------------------------------------------------------

mobility_change | Odds ratio Std. err. z P>|z| [95% conf. interval]

----------------+----------------------------------------------------------------

timeadsu_cat |

<12h | 1.102393 .4107184 0.26 0.794 .5311345 2.288066

<24h | .6121584 .1839047 -1.63 0.102 .3397392 1.103016

> 24h | .8236081 .309547 -0.52 0.606 .3942782 1.720436

|

Age | 1.034866 .0188512 1.88 0.060 .9985698 1.072481

|

sex |

male | 1.654839 .4746577 1.76 0.079 .9432032 2.903394

_cons | .0849874 .1317982 -1.59 0.112 .0040674 1.775805

---------------------------------------------------------------------------------

Note: _cons estimates baseline odds.

Exposure: Time between Admission and Surgery in hours; Outcome: Worsened domicile

Logistic regression Number of obs = 281

LR chi2(5) = 18.00

Prob > chi2 = 0.0029

Log likelihood = -153.00388 Pseudo R2 = 0.0556

---------------------------------------------------------------------------------

domicile_change | Odds ratio Std. err. z P>|z| [95% conf. interval]

----------------+----------------------------------------------------------------

timeadsu_cat |

<12h | 1.018966 .4042832 0.05 0.962 .4682074 2.217588

<24h | 1.019546 .3449872 0.06 0.954 .5252703 1.978931

> 24h | .5948315 .2768728 -1.12 0.264 .2388857 1.481146

|

Age | 1.081793 .0233767 3.64 0.000 1.036932 1.128595

|

sex |

male | 1.72207 .5269399 1.78 0.076 .9453435 3.13698

_cons | .0004025 .000758 -4.15 0.000 .00001 .0161377

---------------------------------------------------------------------------------

Note: _cons estimates baseline odds.

Exposure: Invasivness; Outcome: Mortality inpatient

Logistic regression Number of obs = 281

LR chi2(3) = 24.05

Prob > chi2 = 0.0000

Log likelihood = -72.314599 Pseudo R2 = 0.1426

------------------------------------------------------------------------------

death_inc | Odds ratio Std. err. z P>|z| [95% conf. interval]

-------------+----------------------------------------------------------------

invasivness |

Open | 2.075653 .9537271 1.59 0.112 .8434167 5.108193

Age | 1.151057 .0453765 3.57 0.000 1.065469 1.243519

|

sex |

male | 5.018342 2.385899 3.39 0.001 1.976371 12.74242

_cons | 1.83e-07 6.66e-07 -4.27 0.000 1.49e-10 .0002262

------------------------------------------------------------------------------

Note: _cons estimates baseline odds.

Exposure: Invasivness; Outcome: Mortality at 120 d

Logistic regression Number of obs = 281

LR chi2(3) = 21.08

Prob > chi2 = 0.0001

Log likelihood = -122.55801 Pseudo R2 = 0.0792

------------------------------------------------------------------------------

death120 | Odds ratio Std. err. z P>|z| [95% conf. interval]

-------------+----------------------------------------------------------------

invasivness |

Open | 1.772823 .5864595 1.73 0.083 .9270076 3.390373

Age | 1.094292 .0281307 3.51 0.000 1.040523 1.15084

|

sex |

male | 2.725891 .9358126 2.92 0.003 1.39087 5.342326

_cons | .0000531 .0001221 -4.28 0.000 5.88e-07 .0047974

------------------------------------------------------------------------------

Note: _cons estimates baseline odds.

Exposure: Invasivness; Outcome: Worsened mobility

Logistic regression Number of obs = 281

LR chi2(3) = 12.22

Prob > chi2 = 0.0067

Log likelihood = -183.63721 Pseudo R2 = 0.0322

---------------------------------------------------------------------------------

mobility_change | Odds ratio Std. err. z P>|z| [95% conf. interval]

----------------+----------------------------------------------------------------

invasivness |

Open | .5687365 .1417602 -2.26 0.024 .3489355 .9269941

Age | 1.036033 .0188949 1.94 0.052 .999654 1.073736

|

sex |

male | 1.61844 .4640135 1.68 0.093 .9226852 2.838832

_cons | .0883498 .1368843 -1.57 0.117 .0042403 1.840818

---------------------------------------------------------------------------------

Note: _cons estimates baseline odds.

Exposure: Invasivness; Outcome: Worsened domicile

Logistic regression Number of obs = 281

LR chi2(3) = 16.89

Prob > chi2 = 0.0007

Log likelihood = -153.55945 Pseudo R2 = 0.0521

---------------------------------------------------------------------------------

domicile_change | Odds ratio Std. err. z P>|z| [95% conf. interval]

----------------+----------------------------------------------------------------

invasivness |

Open | 1.222895 .3446966 0.71 0.475 .7038196 2.124793

Age | 1.082598 .0232789 3.69 0.000 1.037921 1.129199

|

sex |

male | 1.74662 .5327912 1.83 0.068 .9606096 3.175777

_cons | .0003192 .000603 -4.26 0.000 7.87e-06 .0129441

---------------------------------------------------------------------------------

Note: _cons estimates baseline odds.

Exposure: Implant; Outcome: Mortality inpatient

note: 3.implant != 0 predicts failure perfectly;

3.implant omitted and 14 obs not used.

Logistic regression Number of obs = 267

LR chi2(3) = 21.27

Prob > chi2 = 0.0001

Log likelihood = -72.365625 Pseudo R2 = 0.1281

-----------------------------------------------------------------------------------

death_inc | Odds ratio Std. err. z P>|z| [95% conf. interval]

------------------+----------------------------------------------------------------

implant |

Nail open or THR | 1.260492 .5660931 0.52 0.606 .5227106 3.039617

Osteosynthesis | 1 (empty)

|

Age | 1.143151 .043628 3.51 0.000 1.060762 1.23194

|

sex |

male | 4.310069 2.006975 3.14 0.002 1.73031 10.73605

_cons | 5.07e-07 1.77e-06 -4.16 0.000 5.52e-10 .0004666

-----------------------------------------------------------------------------------

Note: _cons estimates baseline odds.

Exposure: Implant; Outcome: Mortality at 120 d

Logistic regression Number of obs = 281

LR chi2(4) = 19.27

Prob > chi2 = 0.0007

Log likelihood = -123.46203 Pseudo R2 = 0.0724

-----------------------------------------------------------------------------------

death120 | Odds ratio Std. err. z P>|z| [95% conf. interval]

------------------+----------------------------------------------------------------

implant |

Nail open or THR | 1.403709 .4697386 1.01 0.311 .7285013 2.704731

Osteosynthesis | 1.65118 1.18446 0.70 0.484 .4047546 6.73592

|

Age | 1.090949 .0276583 3.43 0.001 1.038065 1.146528

|

sex |

male | 2.677489 .9188065 2.87 0.004 1.366563 5.24597

_cons | .0000797 .0001795 -4.19 0.000 9.63e-07 .0065961

-----------------------------------------------------------------------------------

Note: _cons estimates baseline odds.

Exposure: Implant; Outcome: Worsened mobility

Logistic regression Number of obs = 281

LR chi2(4) = 16.50

Prob > chi2 = 0.0024

Log likelihood = -181.49433 Pseudo R2 = 0.0435

-----------------------------------------------------------------------------------

mobility_change | Odds ratio Std. err. z P>|z| [95% conf. interval]

------------------+----------------------------------------------------------------

implant |

Nail open or THR | .5242438 .1348318 -2.51 0.012 .3166725 .8678732

Osteosynthesis | .2727878 .1619884 -2.19 0.029 .0851843 .8735553

|

Age | 1.037421 .0191271 1.99 0.046 1.000602 1.075595

|

sex |

male | 1.544241 .4467798 1.50 0.133 .8758815 2.722605

_cons | .0841909 .1316189 -1.58 0.113 .0039314 1.802941

-----------------------------------------------------------------------------------

Note: _cons estimates baseline odds.

Exposure: Implant; Outcome: Worsened domicile

Logistic regression Number of obs = 281

LR chi2(4) = 16.43

Prob > chi2 = 0.0025

Log likelihood = -153.79022 Pseudo R2 = 0.0507

-----------------------------------------------------------------------------------

domicile_change | Odds ratio Std. err. z P>|z| [95% conf. interval]

------------------+----------------------------------------------------------------

implant |

Nail open or THR | 1.019595 .2962432 0.07 0.947 .5769145 1.801956

Osteosynthesis | 1.154831 .7428472 0.22 0.823 .3273264 4.074323

|

Age | 1.08112 .0231331 3.65 0.000 1.036717 1.127424

|

sex |

male | 1.726844 .5285267 1.78 0.074 .9478291 3.146127

_cons | .0003933 .0007365 -4.19 0.000 .00001 .0154348

-----------------------------------------------------------------------------------

Note: _cons estimates baseline odds.

Exposure: BMI in kg/m^2; Outcome: Mortality inpatient

Logistic regression Number of obs = 281

LR chi2(3) = 22.74

Prob > chi2 = 0.0000

Log likelihood = -72.968097 Pseudo R2 = 0.1348

------------------------------------------------------------------------------

death_inc | Odds ratio Std. err. z P>|z| [95% conf. interval]

-------------+----------------------------------------------------------------

bmi | .9280629 .0617281 -1.12 0.262 .8146322 1.057288

Age | 1.130842 .0424489 3.28 0.001 1.050631 1.217178

|

sex |

male | 4.92754 2.344034 3.35 0.001 1.939604 12.51835

_cons | 7.37e-06 .0000286 -3.04 0.002 3.66e-09 .0148535

------------------------------------------------------------------------------

Note: _cons estimates baseline odds.

Exposure: BMI in kg/m^2; Outcome: Mortality at 120 d

Logistic regression Number of obs = 281

LR chi2(3) = 23.20

Prob > chi2 = 0.0000

Log likelihood = -121.49737 Pseudo R2 = 0.0871

------------------------------------------------------------------------------

death120 | Odds ratio Std. err. z P>|z| [95% conf. interval]

-------------+----------------------------------------------------------------

bmi | .8993622 .0432377 -2.21 0.027 .8184879 .9882276

Age | 1.079967 .0268762 3.09 0.002 1.028554 1.133949

|

sex |

male | 2.857942 .9937779 3.02 0.003 1.445671 5.649857

_cons | .0026217 .0065961 -2.36 0.018 .0000189 .3632026

------------------------------------------------------------------------------

Note: _cons estimates baseline odds.

Exposure: BMI in kg/m^2; Outcome: Worsened mobility

Logistic regression Number of obs = 281

LR chi2(3) = 7.05

Prob > chi2 = 0.0703

Log likelihood = -186.22062 Pseudo R2 = 0.0186

---------------------------------------------------------------------------------

mobility_change | Odds ratio Std. err. z P>|z| [95% conf. interval]

----------------+----------------------------------------------------------------

bmi | .9962141 .0318366 -0.12 0.906 .9357296 1.060608

Age | 1.036424 .018902 1.96 0.050 1.000031 1.074141

|

sex |

male | 1.676361 .4763655 1.82 0.069 .9604722 2.925839

_cons | .06905 .125999 -1.46 0.143 .0019317 2.468276

---------------------------------------------------------------------------------

Note: _cons estimates baseline odds.

Exposure: BMI in kg/m^2; Outcome: Worsened domicile

Logistic regression Number of obs = 281

LR chi2(3) = 18.56

Prob > chi2 = 0.0003

Log likelihood = -152.72341 Pseudo R2 = 0.0573

---------------------------------------------------------------------------------

domicile_change | Odds ratio Std. err. z P>|z| [95% conf. interval]

----------------+----------------------------------------------------------------

bmi | .9443478 .037086 -1.46 0.145 .8743877 1.019906

Age | 1.075912 .0230751 3.41 0.001 1.031623 1.122102

|

sex |

male | 1.798527 .5518006 1.91 0.056 .9857397 3.281493

_cons | .0023008 .0049857 -2.80 0.005 .0000329 .1608373

---------------------------------------------------------------------------------

Note: _cons estimates baseline odds.

Exposure: Hemoglobin pre-surgery; g/dL; Outcome: Mortality inpatient

Logistic regression Number of obs = 281

LR chi2(3) = 22.59

Prob > chi2 = 0.0000

Log likelihood = -73.045885 Pseudo R2 = 0.1339

--------------------------------------------------------------------------------

death_inc | Odds ratio Std. err. z P>|z| [95% conf. interval]

---------------+----------------------------------------------------------------

hemoglobin_pre | .8808025 .1025976 -1.09 0.276 .7010176 1.106696

Age | 1.139822 .0431043 3.46 0.001 1.058394 1.227514

|

sex |

male | 4.441399 2.057787 3.22 0.001 1.791189 11.01281

_cons | 3.22e-06 .0000117 -3.47 0.001 2.56e-09 .0040433

--------------------------------------------------------------------------------

Note: _cons estimates baseline odds.

Exposure: Hemoglobin pre-surgery; g/dL; Outcome: Mortality at 120 d

Logistic regression Number of obs = 281

LR chi2(3) = 25.44

Prob > chi2 = 0.0000

Log likelihood = -120.37609 Pseudo R2 = 0.0956

--------------------------------------------------------------------------------

death120 | Odds ratio Std. err. z P>|z| [95% conf. interval]

---------------+----------------------------------------------------------------

hemoglobin_pre | .7868297 .0698344 -2.70 0.007 .6612003 .9363289

Age | 1.090857 .0278643 3.40 0.001 1.037589 1.14686

|

sex |

male | 2.543763 .8746708 2.72 0.007 1.296557 4.990701

_cons | .0018228 .0043723 -2.63 0.009 .0000166 .2006711

--------------------------------------------------------------------------------

Note: _cons estimates baseline odds.

Exposure: Hemoglobin pre-surgery; g/dL; Outcome: Worsened mobility

Logistic regression Number of obs = 281

LR chi2(3) = 12.80

Prob > chi2 = 0.0051

Log likelihood = -183.34445 Pseudo R2 = 0.0337

---------------------------------------------------------------------------------

mobility_change | Odds ratio Std. err. z P>|z| [95% conf. interval]

----------------+----------------------------------------------------------------

hemoglobin_pre | .839522 .0631135 -2.33 0.020 .7245036 .9728001

Age | 1.035479 .0187972 1.92 0.055 .9992846 1.072984

|

sex |

male | 1.663505 .4770268 1.77 0.076 .948273 2.918198

_cons | .60976 1.109219 -0.27 0.786 .0172477 21.55689

---------------------------------------------------------------------------------

Note: _cons estimates baseline odds.

Exposure: Hemoglobin pre-surgery; g/dL; Outcome: Worsened domicile

Logistic regression Number of obs = 281

LR chi2(3) = 21.35

Prob > chi2 = 0.0001

Log likelihood = -151.32667 Pseudo R2 = 0.0659

---------------------------------------------------------------------------------

domicile_change | Odds ratio Std. err. z P>|z| [95% conf. interval]

----------------+----------------------------------------------------------------

hemoglobin_pre | .8402009 .0660147 -2.22 0.027 .7202846 .9800814

Age | 1.081865 .0233834 3.64 0.000 1.036991 1.12868

|

sex |

male | 1.696865 .5206711 1.72 0.085 .9299557 3.096225

_cons | .0032409 .0066976 -2.77 0.006 .0000564 .186104

---------------------------------------------------------------------------------

Note: _cons estimates baseline odds.

Exposure: Hemoglobin minimal; g/dL; Outcome: Mortality inpatient

Logistic regression Number of obs = 281

LR chi2(3) = 25.62

Prob > chi2 = 0.0000

Log likelihood = -71.531611 Pseudo R2 = 0.1519

--------------------------------------------------------------------------------

death_inc | Odds ratio Std. err. z P>|z| [95% conf. interval]

---------------+----------------------------------------------------------------

hemoglobin_min | .7224618 .1201738 -1.95 0.051 .5214667 1.000929

Age | 1.139799 .043881 3.40 0.001 1.056958 1.229132

|

sex |

male | 5.222295 2.507506 3.44 0.001 2.037762 13.38348

_cons | 8.47e-06 .0000309 -3.20 0.001 6.57e-09 .0109127

--------------------------------------------------------------------------------

Note: _cons estimates baseline odds.

Exposure: Hemoglobin minimal; g/dL; Outcome: Mortality at 120 d

Logistic regression Number of obs = 281

LR chi2(3) = 19.73

Prob > chi2 = 0.0002

Log likelihood = -123.23353 Pseudo R2 = 0.0741

--------------------------------------------------------------------------------

death120 | Odds ratio Std. err. z P>|z| [95% conf. interval]

---------------+----------------------------------------------------------------

hemoglobin_min | .8627916 .0988415 -1.29 0.198 .6892744 1.07999

Age | 1.08597 .0273082 3.28 0.001 1.033745 1.140834

|

sex |

male | 2.669337 .9120315 2.87 0.004 1.366387 5.214746

_cons | .0004663 .0011309 -3.16 0.002 4.02e-06 .0540735

--------------------------------------------------------------------------------

Note: _cons estimates baseline odds.

Exposure: Hemoglobin minimal; g/dL; Outcome: Worsened mobility

Logistic regression Number of obs = 281

LR chi2(3) = 9.20

Prob > chi2 = 0.0268

Log likelihood = -185.1482 Pseudo R2 = 0.0242

---------------------------------------------------------------------------------

mobility_change | Odds ratio Std. err. z P>|z| [95% conf. interval]

----------------+----------------------------------------------------------------

hemoglobin_min | .8835823 .074686 -1.46 0.143 .7486831 1.042788

Age | 1.032755 .0188225 1.77 0.077 .9965151 1.070314

|

sex |

male | 1.708978 .4871729 1.88 0.060 .9774332 2.988035

_cons | .2357617 .4188522 -0.81 0.416 .0072483 7.668509

---------------------------------------------------------------------------------

Note: _cons estimates baseline odds.

Exposure: Hemoglobin minimal; g/dL; Outcome: Worsened domicile

Logistic regression Number of obs = 281

LR chi2(3) = 17.89

Prob > chi2 = 0.0005

Log likelihood = -153.06044 Pseudo R2 = 0.0552

---------------------------------------------------------------------------------

domicile_change | Odds ratio Std. err. z P>|z| [95% conf. interval]

----------------+----------------------------------------------------------------

hemoglobin_min | .8864979 .0879757 -1.21 0.225 .7298016 1.076839

Age | 1.078366 .023162 3.51 0.000 1.033911 1.124732

|

sex |

male | 1.770794 .5413251 1.87 0.062 .9726549 3.223867

_cons | .0013186 .002748 -3.18 0.001 .0000222 .0783536

---------------------------------------------------------------------------------

Note: _cons estimates baseline odds.

Exposure: Transfusion; Outcome: Mortality inpatient

Logistic regression Number of obs = 281

LR chi2(3) = 21.81

Prob > chi2 = 0.0001

Log likelihood = -73.433092 Pseudo R2 = 0.1293

------------------------------------------------------------------------------

death_inc | Odds ratio Std. err. z P>|z| [95% conf. interval]

-------------+----------------------------------------------------------------

transfusion |

Yes | 1.316345 .5868563 0.62 0.538 .5493953 3.153948

Age | 1.13879 .0430617 3.44 0.001 1.057443 1.226396

|

sex |

male | 4.624308 2.15768 3.28 0.001 1.85302 11.5402

_cons | 6.43e-07 2.20e-06 -4.16 0.000 7.81e-10 .000529

------------------------------------------------------------------------------

Note: _cons estimates baseline odds.

Exposure: Transfusion; Outcome: Mortality at 120 d

Logistic regression Number of obs = 281

LR chi2(3) = 21.99

Prob > chi2 = 0.0001

Log likelihood = -122.10212 Pseudo R2 = 0.0826

------------------------------------------------------------------------------

death120 | Odds ratio Std. err. z P>|z| [95% conf. interval]

-------------+----------------------------------------------------------------

transfusion |

Yes | 1.913362 .6224896 1.99 0.046 1.011274 3.620141

Age | 1.086545 .0274887 3.28 0.001 1.033982 1.14178

|

sex |

male | 2.702335 .9278565 2.90 0.004 1.37872 5.296662

_cons | .0001008 .0002247 -4.13 0.000 1.28e-06 .0079639

------------------------------------------------------------------------------

Note: _cons estimates baseline odds.

Exposure: Transfusion; Outcome: Worsened mobility

Logistic regression Number of obs = 281

LR chi2(3) = 9.27

Prob > chi2 = 0.0259

Log likelihood = -185.11282 Pseudo R2 = 0.0244

---------------------------------------------------------------------------------

mobility_change | Odds ratio Std. err. z P>|z| [95% conf. interval]

----------------+----------------------------------------------------------------

transfusion |

Yes | 1.467315 .3788081 1.49 0.137 .8846545 2.433734

Age | 1.034183 .0187355 1.86 0.064 .9981063 1.071563

|

sex |

male | 1.698496 .483628 1.86 0.063 .972063 2.967801

_cons | .0653708 .0999079 -1.78 0.074 .0032694 1.307065

---------------------------------------------------------------------------------

Note: _cons estimates baseline odds.

Exposure: Transfusion; Outcome: Worsened domicile

Logistic regression Number of obs = 281

LR chi2(3) = 19.18

Prob > chi2 = 0.0003

Log likelihood = -152.41451 Pseudo R2 = 0.0592

---------------------------------------------------------------------------------

domicile_change | Odds ratio Std. err. z P>|z| [95% conf. interval]

----------------+----------------------------------------------------------------

transfusion |

Yes | 1.609898 .4570288 1.68 0.093 .9228973 2.808298

Age | 1.078954 .0231717 3.54 0.000 1.034481 1.125339

|

sex |

male | 1.772223 .5424071 1.87 0.062 .9727459 3.228772

_cons | .0003862 .0007208 -4.21 0.000 9.96e-06 .014978

---------------------------------------------------------------------------------

Note: _cons estimates baseline odds.

Exposure: Amount Of Transfusion; Outcome: Mortality inpatient

Logistic regression Number of obs = 107

LR chi2(3) = 8.34

Prob > chi2 = 0.0394

Log likelihood = -31.266203 Pseudo R2 = 0.1177

------------------------------------------------------------------------------

death_inc | Odds ratio Std. err. z P>|z| [95% conf. interval]

-------------+----------------------------------------------------------------

amount | 1.282153 .4156356 0.77 0.443 .6792128 2.420326

Age | 1.143217 .0692881 2.21 0.027 1.015171 1.287414

|

sex |

male | 4.346263 3.1194 2.05 0.041 1.064613 17.74354

_cons | 3.86e-07 2.18e-06 -2.61 0.009 5.95e-12 .0250482

------------------------------------------------------------------------------

Note: _cons estimates baseline odds.

Exposure: Amount Of Transfusion; Outcome: Mortality at 120 d

Logistic regression Number of obs = 107

LR chi2(3) = 19.30

Prob > chi2 = 0.0002

Log likelihood = -49.681457 Pseudo R2 = 0.1627

------------------------------------------------------------------------------

death120 | Odds ratio Std. err. z P>|z| [95% conf. interval]

-------------+----------------------------------------------------------------

amount | 1.7731 .4240437 2.39 0.017 1.109597 2.833357

Age | 1.166367 .0552773 3.25 0.001 1.062905 1.2799

|

sex |

male | 4.249739 2.491694 2.47 0.014 1.346754 13.41023

_cons | 1.14e-07 5.05e-07 -3.60 0.000 1.90e-11 .000682

------------------------------------------------------------------------------

Note: _cons estimates baseline odds.

Exposure: Amount Of Transfusion; Outcome: Worsened mobility

Logistic regression Number of obs = 107

LR chi2(3) = 4.88

Prob > chi2 = 0.1808

Log likelihood = -66.554394 Pseudo R2 = 0.0354

---------------------------------------------------------------------------------

mobility_change | Odds ratio Std. err. z P>|z| [95% conf. interval]

----------------+----------------------------------------------------------------

amount | 1.373972 .3133765 1.39 0.164 .8786892 2.148427

Age | 1.019452 .0317639 0.62 0.536 .9590588 1.083648

|

sex |

male | 2.38022 1.270106 1.63 0.104 .8363895 6.773696

_cons | .1686155 .4661036 -0.64 0.520 .000748 38.00785

---------------------------------------------------------------------------------

Note: _cons estimates baseline odds.

Exposure: Amount Of Transfusion; Outcome: Worsened domicile

Logistic regression Number of obs = 107

LR chi2(3) = 21.22

Prob > chi2 = 0.0001

Log likelihood = -57.023479 Pseudo R2 = 0.1569

---------------------------------------------------------------------------------

domicile_change | Odds ratio Std. err. z P>|z| [95% conf. interval]

----------------+----------------------------------------------------------------

amount | 1.56734 .3506061 2.01 0.045 1.011006 2.429813

Age | 1.177573 .0513042 3.75 0.000 1.081192 1.282545

|

sex |

male | 2.892648 1.590629 1.93 0.053 .9845344 8.498851

_cons | 1.17e-07 4.70e-07 -3.97 0.000 4.40e-11 .0003105

---------------------------------------------------------------------------------

Note: _cons estimates baseline odds.

Exposure: Antibiotics; Outcome: Mortality inpatient

Logistic regression Number of obs = 281

LR chi2(3) = 21.61

Prob > chi2 = 0.0001

Log likelihood = -73.536787 Pseudo R2 = 0.1281

------------------------------------------------------------------------------

death_inc | Odds ratio Std. err. z P>|z| [95% conf. interval]

-------------+----------------------------------------------------------------

antibiotics |

Yes | .8295282 .3783385 -0.41 0.682 .3393186 2.027938

Age | 1.139806 .0429675 3.47 0.001 1.058628 1.22721

|

sex |

male | 4.45992 2.062676 3.23 0.001 1.801579 11.04081

_cons | 7.27e-07 2.47e-06 -4.15 0.000 9.24e-10 .0005719

------------------------------------------------------------------------------

Note: _cons estimates baseline odds.

Exposure: Antibiotics; Outcome: Mortality at 120 d

Logistic regression Number of obs = 281

LR chi2(3) = 18.81

Prob > chi2 = 0.0003

Log likelihood = -123.69225 Pseudo R2 = 0.0707

------------------------------------------------------------------------------

death120 | Odds ratio Std. err. z P>|z| [95% conf. interval]

-------------+----------------------------------------------------------------

antibiotics |

Yes | 1.336752 .4330009 0.90 0.370 .7084836 2.522158

Age | 1.087789 .0270981 3.38 0.001 1.035953 1.142218

|

sex |

male | 2.576287 .8716923 2.80 0.005 1.327359 5.000347

_cons | .0001092 .0002392 -4.16 0.000 1.49e-06 .0080089

------------------------------------------------------------------------------

Note: _cons estimates baseline odds.

Exposure: Antibiotics; Outcome: Worsened mobility

Logistic regression Number of obs = 281

LR chi2(3) = 7.59

Prob > chi2 = 0.0552

Log likelihood = -185.9495 Pseudo R2 = 0.0200

---------------------------------------------------------------------------------

mobility_change | Odds ratio Std. err. z P>|z| [95% conf. interval]

----------------+----------------------------------------------------------------

antibiotics |

Yes | 1.207273 .3054566 0.74 0.457 .7352584 1.982308

Age | 1.036484 .0186873 1.99 0.047 1.000497 1.073766

|

sex |

male | 1.681291 .4775474 1.83 0.067 .9635429 2.933695

_cons | .0582624 .0889969 -1.86 0.063 .0029185 1.163092

---------------------------------------------------------------------------------

Note: _cons estimates baseline odds.

Exposure: Antibiotics; Outcome: Worsened domicile

Logistic regression Number of obs = 281

LR chi2(3) = 18.00

Prob > chi2 = 0.0004

Log likelihood = -153.00345 Pseudo R2 = 0.0556

---------------------------------------------------------------------------------

domicile_change | Odds ratio Std. err. z P>|z| [95% conf. interval]

----------------+----------------------------------------------------------------

antibiotics |

Yes | 1.43488 .4058149 1.28 0.202 .8242854 2.497777

Age | 1.080658 .0230566 3.64 0.000 1.0364 1.126806

|

sex |

male | 1.737829 .5292229 1.81 0.070 .9567308 3.156635

_cons | .000356 .0006611 -4.28 0.000 9.35e-06 .0135571

---------------------------------------------------------------------------------

Note: _cons estimates baseline odds.

Exposure: Dementia; Outcome: Mortality inpatient

Logistic regression Number of obs = 279

LR chi2(4) = 23.98

Prob > chi2 = 0.0001

Log likelihood = -69.820438 Pseudo R2 = 0.1466

------------------------------------------------------------------------------

death_inc | Odds ratio Std. err. z P>|z| [95% conf. interval]

-------------+----------------------------------------------------------------

dementia_del |

Dementia | .6530918 .3301507 -0.84 0.399 .2424796 1.75903

Delerium | .1590459 .1756234 -1.67 0.096 .0182641 1.384987

|

Age | 1.148754 .0442332 3.60 0.000 1.065249 1.238805

|

sex |

male | 4.269301 2.040268 3.04 0.002 1.6733 10.89281

_cons | 5.16e-07 1.76e-06 -4.23 0.000 6.34e-10 .0004196

------------------------------------------------------------------------------

Note: _cons estimates baseline odds.

Exposure: Dementia; Outcome: Mortality at 120 d

Logistic regression Number of obs = 279

LR chi2(4) = 16.86

Prob > chi2 = 0.0021

Log likelihood = -122.75705 Pseudo R2 = 0.0642

------------------------------------------------------------------------------

death120 | Odds ratio Std. err. z P>|z| [95% conf. interval]

-------------+----------------------------------------------------------------

dementia_del |

Dementia | .9237233 .3589196 -0.20 0.838 .4313216 1.978256

Delerium | .9171902 .4951457 -0.16 0.873 .3183741 2.642294

|

Age | 1.088985 .027901 3.33 0.001 1.035651 1.145066

|

sex |

male | 2.399089 .8217622 2.55 0.011 1.22598 4.694715

_cons | .00012 .0002648 -4.09 0.000 1.59e-06 .0090749

------------------------------------------------------------------------------

Note: _cons estimates baseline odds.

Exposure: Dementia; Outcome: Worsened mobility

Logistic regression Number of obs = 279

LR chi2(4) = 6.27

Prob > chi2 = 0.1802

Log likelihood = -185.19037 Pseudo R2 = 0.0166

---------------------------------------------------------------------------------

mobility_change | Odds ratio Std. err. z P>|z| [95% conf. interval]

----------------+----------------------------------------------------------------

dementia_del |

Dementia | .951743 .2773591 -0.17 0.865 .5376016 1.684918

Delerium | 1.108339 .4724113 0.24 0.809 .4806863 2.555544

|

Age | 1.034772 .0193731 1.83 0.068 .9974901 1.073448

|

sex |

male | 1.608004 .4597096 1.66 0.097 .9182022 2.81602

_cons | .0742047 .1147203 -1.68 0.092 .0035849 1.535987

---------------------------------------------------------------------------------

Note: _cons estimates baseline odds.

Exposure: Dementia; Outcome: Worsened domicile

Logistic regression Number of obs = 279

LR chi2(4) = 15.62

Prob > chi2 = 0.0036

Log likelihood = -152.55146 Pseudo R2 = 0.0487

---------------------------------------------------------------------------------

domicile_change | Odds ratio Std. err. z P>|z| [95% conf. interval]

----------------+----------------------------------------------------------------

dementia_del |

Dementia | 1.149644 .393422 0.41 0.684 .5878577 2.248303

Delerium | 1.075385 .5139369 0.15 0.879 .4214697 2.743857

|

Age | 1.078418 .0236424 3.44 0.001 1.033061 1.125766

|

sex |

male | 1.655694 .5098653 1.64 0.102 .9054314 3.027643

_cons | .0004512 .0008431 -4.12 0.000 .0000116 .0175781

---------------------------------------------------------------------------------

Note: _cons estimates baseline odds.

Exposure: Nutritional risk score; Outcome: Mortality inpatient

Logistic regression Number of obs = 185

LR chi2(3) = 18.41

Prob > chi2 = 0.0004

Log likelihood = -45.246105 Pseudo R2 = 0.1691

------------------------------------------------------------------------------

death_inc | Odds ratio Std. err. z P>|z| [95% conf. interval]

-------------+----------------------------------------------------------------

nutri_scorek |

>=3 | 6.694199 5.356566 2.38 0.018 1.395031 32.1228

Age | 1.120811 .0546979 2.34 0.019 1.018572 1.233311

|

sex |

male | 4.893954 3.000177 2.59 0.010 1.471775 16.2734

_cons | 6.67e-07 2.99e-06 -3.17 0.002 1.01e-10 .0044222

------------------------------------------------------------------------------

Note: _cons estimates baseline odds.

Exposure: Nutritional risk score; Outcome: Mortality at 120 d

Logistic regression Number of obs = 185

LR chi2(3) = 20.73

Prob > chi2 = 0.0001

Log likelihood = -76.385428 Pseudo R2 = 0.1195

------------------------------------------------------------------------------

death120 | Odds ratio Std. err. z P>|z| [95% conf. interval]

-------------+----------------------------------------------------------------

nutri_scorek |

>=3 | 3.2959 1.565382 2.51 0.012 1.299264 8.360858

Age | 1.1107 .0375107 3.11 0.002 1.039561 1.186707

|

sex |

male | 2.235881 .994336 1.81 0.070 .9351992 5.345559

_cons | 9.14e-06 .0000275 -3.86 0.000 2.51e-08 .0033232

------------------------------------------------------------------------------

Note: _cons estimates baseline odds.

Exposure: Nutritional risk score; Outcome: Worsened mobility

Logistic regression Number of obs = 185

LR chi2(3) = 5.38

Prob > chi2 = 0.1459

Log likelihood = -118.97401 Pseudo R2 = 0.0221

---------------------------------------------------------------------------------

mobility_change | Odds ratio Std. err. z P>|z| [95% conf. interval]

----------------+----------------------------------------------------------------

nutri_scorek |

>=3 | 1.439623 .4513923 1.16 0.245 .7786735 2.661596

Age | 1.035916 .0247097 1.48 0.139 .9886004 1.085496

|

sex |

male | 1.607876 .5676049 1.35 0.179 .8049433 3.211735

_cons | .0627916 .1263794 -1.38 0.169 .0012154 3.244126

---------------------------------------------------------------------------------

Note: _cons estimates baseline odds.

Exposure: Nutritional risk score; Outcome: Worsened domicile

Logistic regression Number of obs = 185

LR chi2(3) = 19.35

Prob > chi2 = 0.0002

Log likelihood = -95.17039 Pseudo R2 = 0.0923

---------------------------------------------------------------------------------

domicile_change | Odds ratio Std. err. z P>|z| [95% conf. interval]

----------------+----------------------------------------------------------------

nutri_scorek |

>=3 | 2.682438 1.045772 2.53 0.011 1.249342 5.759411

Age | 1.090837 .0312709 3.03 0.002 1.031237 1.153881

|

sex |

male | 1.828419 .7105311 1.55 0.120 .8536808 3.916121

_cons | .0000899 .0002257 -3.71 0.000 6.55e-07 .0123411

---------------------------------------------------------------------------------

Note: _cons estimates baseline odds.
